# Long‐Term Clinical Outcomes of Transurethral Resection of Hunner Lesions Combined With Bladder Hydrodistension for Patients With Interstitial Cystitis at a Tertiary Referral Center in Japan

**DOI:** 10.1111/iju.70227

**Published:** 2025-09-14

**Authors:** Yoshiyuki Akiyama, Kenichi Hashimoto, Aya Niimi, Yukio Homma, Haruki Kume

**Affiliations:** ^1^ Department of Urology Shinshu University School of Medicine Nagano Japan; ^2^ Department of Urology, Graduate School of Medicine The University of Tokyo Tokyo Japan; ^3^ Department of Interstitial Cystitis Medicine Kyorin University School of Medicine Tokyo Japan

**Keywords:** bladder pain syndrome, endoscopic, fulguration, Hunner, IC, IC/BPS, interstitial cystitis, TUC, TUR

## Abstract

**Objectives:**

To report the long‐term clinical outcomes of transurethral resection of Hunner lesions with bladder hydrodistension in patients with Hunner lesion interstitial cystitis (HIC) at a tertiary referral center in Japan.

**Methods:**

A retrospective chart review was conducted to evaluate the treatment outcomes of 104 patients with HIC who underwent initial endoscopic surgery, including transurethral resection of Hunner lesions (ablation was kept to a minimum) and concomitant bladder hydrodistension between 2017 and 2023. Clinical outcomes were evaluated and compared between each follow‐up visit and baseline over 12 months using a 7‐graded global response assessment (GRA), O'Leary and Sant's symptom and problem indices (OSSI/OSPI), an 11‐point pain intensity numerical rating scale, quality of life (QOL) score, and frequency volume chart variables. Patients with GRA scores ≥ +2 (moderately/marked improved) and scores ≤ −1 (slightly/moderately/marked worse) were considered treatment responders and failures, respectively. Postoperative complications were also documented.

**Results:**

The mean duration to treatment failure was 30.9 ± 21.0 months. The overall response rates at 1, 3, 6, 9, and 12 months were 78.8%, 80.8%, 76.0%, 69.2%, and 57.7%, respectively. Compared with the baseline, the OSSI/OSPI, pain intensity, QOL score, urinary frequency, and functional bladder capacity improved significantly after 1 month and were maintained over 12 months. One patient required a second surgery for postoperative bleeding and another developed distal urethral stenosis 6 months post‐surgery. No other patients developed any postoperative complications.

**Conclusions:**

Transurethral resection of Hunner lesions combined with bladder hydrodistension offers long‐term symptom relief without serious adverse events for treatment‐naïve patients with HIC.

**Abbreviations**
DMSO: dimethyl sulfoxide
GRA: global response assessment
HIC: Hunner lesion interstitial cystitis
IC/BPS: interstitial cystitis/bladder pain syndrome
OSSI/OSPI: O'Leary and Sant symptom index/O'Leary and Sant problem index
QOL: quality of life

## 1 Introduction

Interstitial cystitis/bladder pain syndrome (IC/BPS) is an intractable, debilitating urological disorder, clinically characterized by chronic bladder/pelvic pain in conjunction with lower urinary tract symptoms, such as urinary frequency and urgency [1]. Currently, IC/BPS is subdivided into two subtypes, Hunner lesion IC (HIC) and BPS, based on the presence or absence of Hunner lesions [1, 2]. Evidence shows that HIC and BPS are derived from distinct pathogenesis; HIC is an immune‐mediated inflammatory disease of the urinary bladder with possible autoimmune nature, while BPS is histologically a noninflammatory disease with little evidence of bladder pathology and potentially associated with systemic neurophysiological dysregulation [3, 4, 5, 6, 7, 8]. Thus, treatment strategies should be given in a subtype‐specific manner for HIC and BPS. Today, electrocautery of Hunner lesions, intravesical injection of triamcinolone/dimethyl sulfoxide (DMSO), and systemic immunomodulatory therapies using corticosteroids, cyclosporine, and tacrolimus are used as the standard treatments for HIC [9, 10, 11, 12, 13, 14]. Above all, endoscopic elimination of Hunner lesions, with or without simultaneous bladder hydrodistension, is regarded as the most effective, promising treatment option for patients with HIC, with the reported duration of treatment efficacy ranging from 12 to 28.5 months [9, 15, 16, 17, 18, 19]. In the present study, we evaluated the long‐term clinical outcomes of transurethral resection of Hunner lesions with simultaneous bladder hydrodistension performed by a single expert urologist for treatment‐naïve 104 Japanese patients with HIC at the highest‐volume center of IC/BPS in Japan. Herein, we report that transurethral resection with concomitant bladder hydrodistension significantly ameliorated irritable pain and voiding symptoms, increased bladder capacity, and improved the quality of life (QOL) in patients with HIC without any serious adverse events, achieving long‐term treatment efficacy lasting over 30 months.

## 2 Methods

### 2.1 Ethics Statement

The Institutional Review Board of the University of Tokyo approved the study protocol, including the use of an opt‐out methodology to obtain informed consent (approval no. 3124). The patients were informed about the study design using generally accessible contact information, and written informed consent was obtained from those who chose to participate. All procedures followed the appropriate guidelines.

### 2.2 Patients

This was a retrospective observational study of a prospectively maintained database of 104 patients with HIC who underwent transurethral resection of Hunner lesions with simultaneous bladder hydrodistension as an initial treatment for the disease between 2017 and 2023. Patients presenting with lower urinary tract symptoms and bladder/urethral pain refractory to pharmacological treatments—including anticholinergic agents, alpha‐1 adrenoceptor antagonists, beta‐3 adrenoceptor agonists, antibiotics, or herbal medicines—were referred to our hospital from local urology clinics for suspicion of having IC/BPS. All patients received additional symptomatic care for their intractable bladder pain and discomfort prior to undergoing surgery by oral analgesia, serotonin‐norepinephrine reuptake inhibitors, or tricyclic antidepressants. A tentative diagnosis of HIC was made in our outpatient clinic following the East Asian clinical guidelines [2], and the final diagnosis of HIC was determined after surgery by examining the histological findings of bladder mucosal biopsies obtained at the time of surgery, which were consistent with the characteristic histological features of HIC, namely predominant lymphoplasmacytic infiltration that outnumbered granulocytes, frequent formation of lymph follicles/aggregates, stromal edema/fibrosis, and epithelial denudation [20].

### 2.3 Endoscopic Surgery

**FIGURE 1:**
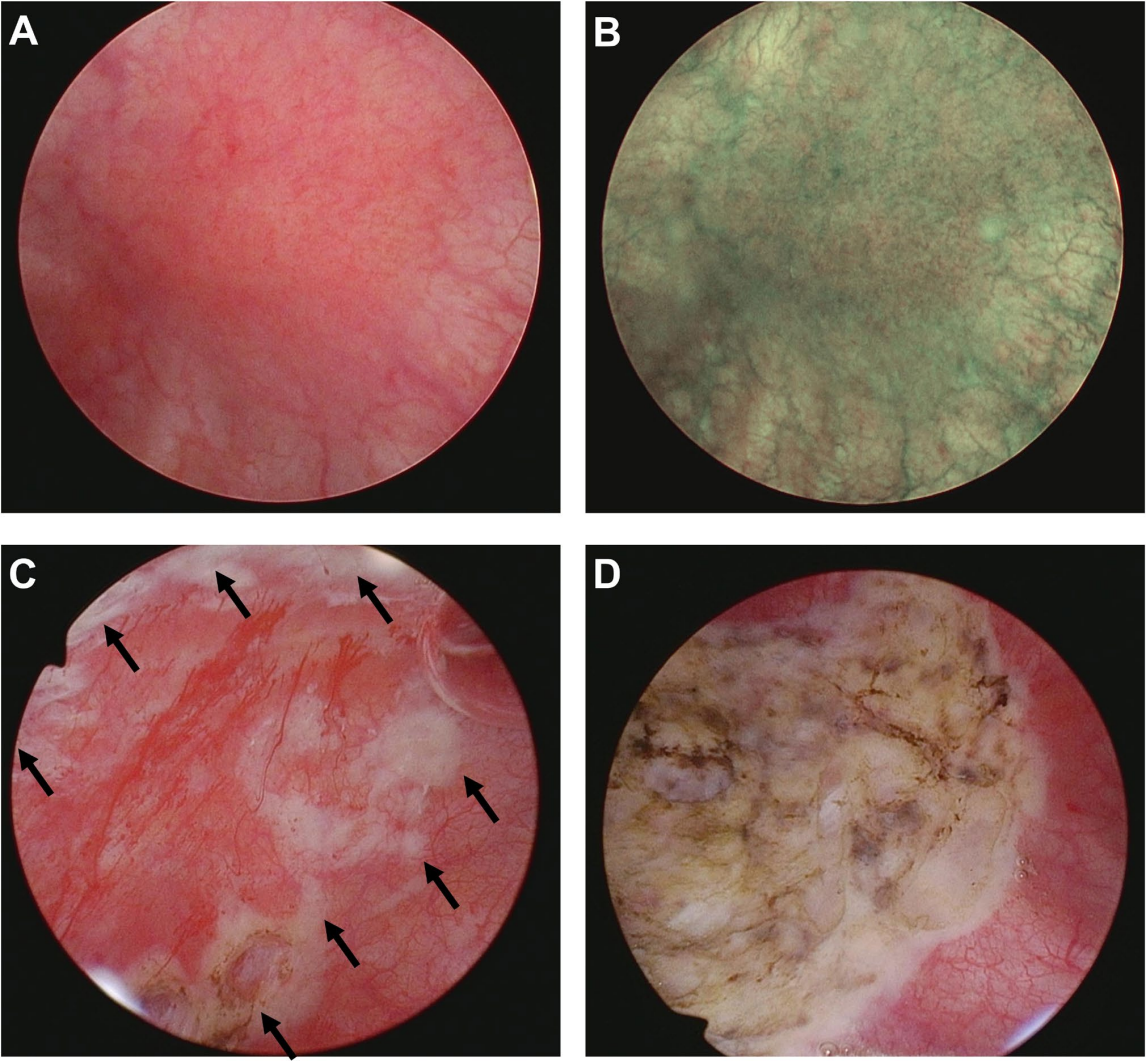
Transurethral resection of Hunner lesion. (A) Hunner lesion observed in posterior wall. (B) Narrow band imaging cystoscopy of the Hunner lesion. (C) Circumferential marking of the Hunner lesion. (D) Transurethral resection of the Hunner lesion.

Eligible patients with HIC underwent endoscopic surgery, including transurethral resection of Hunner lesions combined with bladder hydrodistension, under general anesthesia. During surgery, Hunner lesions were carefully examined, with a minimum amount of normal saline infused into the bladder. The identified Hunner lesions were electrically marked circumferentially to prevent excessive treatment of the bladder mucosa beyond the Hunner lesions at resection. The bladder mucosa was biopsied at both the Hunner lesion and non‐lesion background areas. Subsequently, the bladder was distended with normal saline to the maximum capacity at an intravesical pressure of 80 cm H_2_O for 3 min and then emptied. Maximum bladder capacity (MBC) was measured by collecting the total volume of drained saline after hydrodistension. The location and extent of Hunner lesions, MBC, and post‐distension mucosal bleeding were documented as described previously [21]. Finally, all Hunner lesions were electrically resected along with circumferential marking lines with minimum pinpoint fulguration at the bleeding sites (Figure 1). Postoperative prophylactic antibiotics and acetaminophen were administered for 1 week following surgery. No further treatments, including oral analgesics, intravesical therapies, or additional antibiotics, were provided until symptom recurrence. All surgeries were performed by a single experienced surgeon (YA) in accordance with the aforementioned standardized protocols at our institution. The patients attended follow‐up visits 1 month post‐surgery and then every 3 months thereafter. Peri/postoperative complications and adverse events related to the surgical procedures were carefully monitored during the hospital stay and at every follow‐up visit.

### 2.4 Outcome Assessment

Treatment outcomes were evaluated 1, 3, 6, 9, and 12 months after surgery. Despite the mean duration of efficacy being over a year (30 months), the full assessment of clinical outcomes was completed for up to 12 months because the number of patients who maintained treatment efficacy decreased gradually after 12 months. Treatment response was evaluated using a global response assessment (GRA) questionnaire, which is a 7‐point symmetric scale: markedly improved (+3), moderately improved (+2), slightly improved (+1), no change (0), slightly worse (−1), moderately worse (−2), and markedly worse (−3). Patients who rated treatment efficacy as better than +2 on the GRA were considered responders. Treatment failure was defined at the time of symptom relapse, which was defined by the GRA scores worse than −1. At every follow‐up visit, symptoms were evaluated using the IC/BPS symptom scores measured using O'Leary and Sant's symptom index and problem index (OSSI/PI), an 11‐point numerical rating of pain intensity, with 0 indicating no pain and 10 indicating the worst pain ever, and a seven‐grade QOL scale derived from the International Prostate Symptom Score, with 0 indicating excellent and 6 indicating terrible. Daytime and nocturnal urinary frequency, maximum voided volume (MVV), and average voided volume (AVV) were documented using a frequency volume chart. Demographic information, including age at surgery, duration of illness, MBC status, and previous treatment, was also documented.

### 2.5 Statistical Analysis

Multiple comparisons between the symptom parameters at each follow‐up visit and at baseline were evaluated using the Friedman test, followed by a two‐tailed pairwise comparison using the Wilcoxon signed‐rank test with post hoc Bonferroni correction. A Kaplan–Meier curve was constructed to estimate the duration of symptom relapse after surgery. Logistic regression analysis of the baseline characteristics was applied to identify factors predictive of treatment response at 12 months. Statistical significance was set at *p* < 0.05 (or 0.0033 for multiple comparisons). All statistical analyses were performed using JMP (version 14) (SAS Institute, Cary, NC, USA). Data are expressed as the mean ± standard deviation (SD).

## 3 Results

### 3.1 Patients

The study included 104 patients with an average age of 66.4 ± 12.5 years, consisting of 79 females and 25 males. The demographic and baseline characteristics of the patients are shown in Table 1. All patients were untreated (*N* = 4) or received symptomatic care using oral medicines as listed in Table 1 and had not undergone any endoscopic surgeries/treatments until surgery.

**TABLE 1:** Demographic and baseline characteristics of the study subjects.

No. (male/female)	104 (25/79)
Mean age (years)	66.4 ± 12.5 [20–89]^a^
Duration of illness (years)	3.3 ± 2.4 [0–9]
OSSI	15.2 ± 3.6 [5–20]
OSPI	13.0 ± 3.0 [4–16]
Pain intensity^b^	7.6 ± 2.2 [1–10]
QOL score^c^	5.7 ± 0.6 [4–6]
Daytime frequency	15.1 ± 7.4 [5–19]
Nocturia	4.8 ± 2.8 [0–6]
Average voided volume (mL)	104.3 ± 48.0 [20–250]
Maximum voided volume (mL)	166.4 ± 79.7 [50–490]
Maximum bladder capacity at hydrodistension (mL)	433.4 ± 170.5 [90–1000]
Medicines (no. of patients)	
Nonsteroidal anti‐inflammatory drugs	40
Acetaminophen	14
Opioids	14
Anticholinergic agents	16
Beta‐3 adrenoceptor agonists	27
Alpha‐1 adrenoceptor antagonists	10
Tricyclic antidepressant	16
Serotonin and noradrenaline reuptake inhibitor.	7
Pregabalin	12
Suplatast tosilate	28
PDE5 inhibitor	5
Benzodiazepine	5
Antibiotic agents	10
Herbal medicine	14

Abbreviations: OSSI/OSPI, O'Leary and Sant symptom index/O'Leary and Sant problem index; PDE5, phosphodiesterase type 5; QOL, quality of life.

^a^ Mean ± SD [range].

^b^ Assessed using an 11‐point pain intensity numerical rating scale from 0 (“no pain”) to 10 (“the worst pain ever”).

^c^ Assessed on a 7‐grade quality of life (QOL) scale derived from the International Prostate Symptom Score, with 0 indicating “excellent” and 6 indicating “terrible.”

### 3.2 Long‐Term Treatment Outcomes of Endoscopic Surgery

**FIGURE 2:**
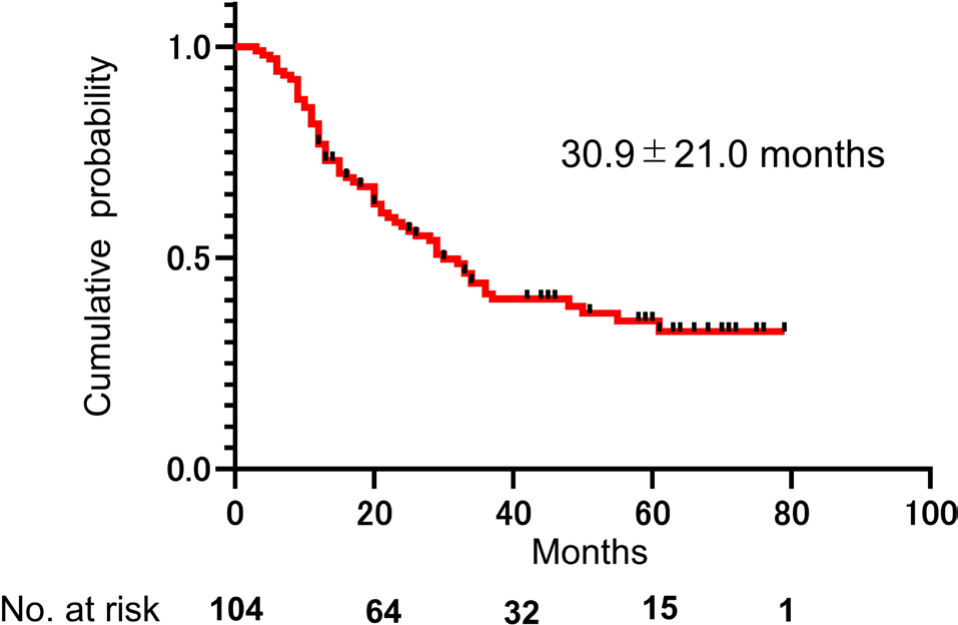
Kaplan–Meier curve for symptom relapse after transurethral resection of Hunner lesions combined with bladder hydrodistension. The duration of symptom relapse after endoscopic surgery was calculated using Kaplan–Meier curves. Symptom relapse was defined as patients rating their condition as worse than −1 (slightly worse) on the GRA. Duration of symptom relapse was defined as the period between surgery and symptom relapse.

The mean duration to treatment failure was 30.9 ± 21.0 months (range, 3–79 months) (Figure 2).

**FIGURE 3:**
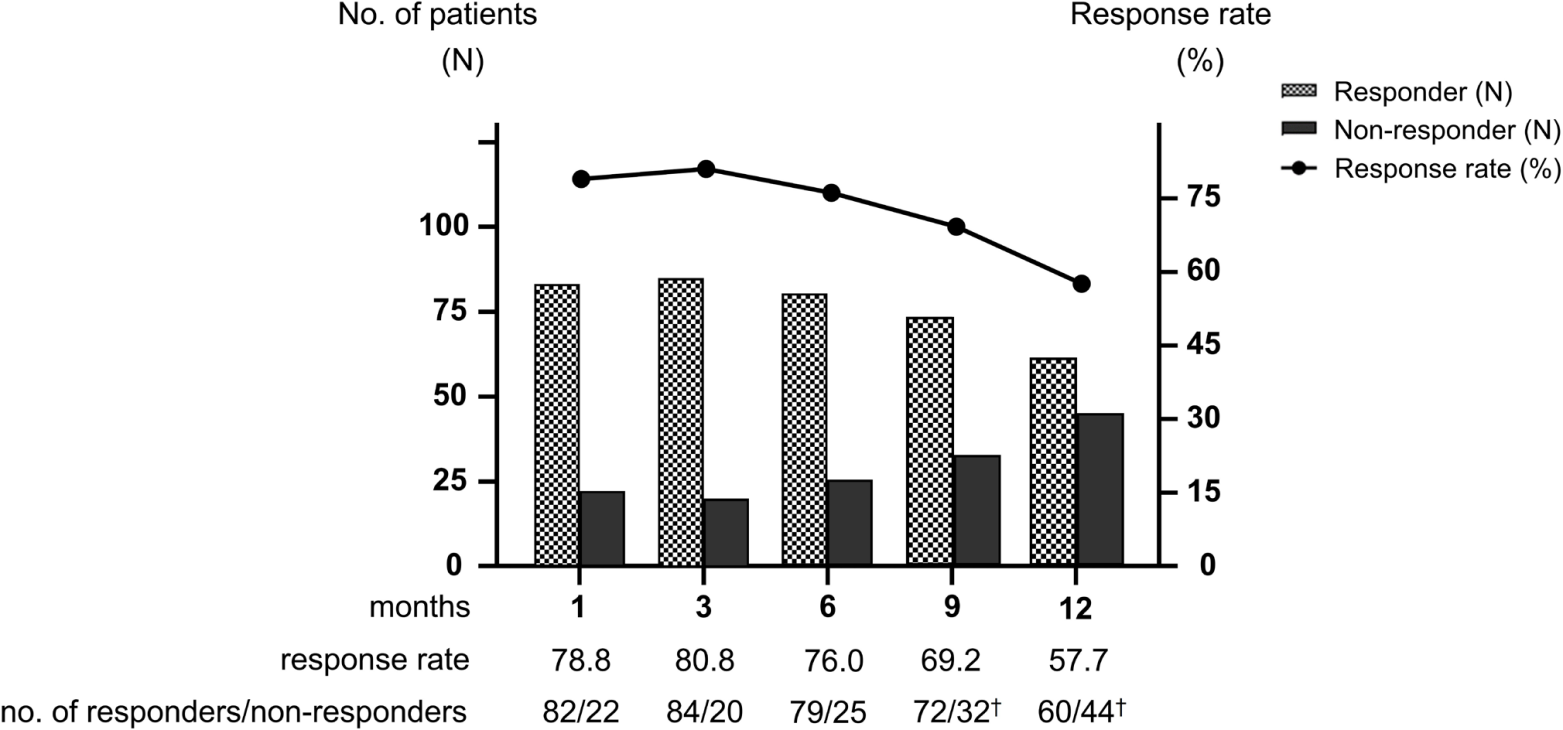
Overall response rates after endoscopic surgery. Treatment response was evaluated using a 7‐graded GRA questionnaire (markedly improved (+3), moderately improved (+2), slightly improved (+1), no change (0), slightly worse (−1), moderately worse (−2), and markedly worse (−3)). Patients who rated the efficacy as better than +2 on the GRA were defined as responders. ^†^Five patients who relapsed and received subsequent treatment within 12 months were included as nonresponders.

**FIGURE 4:**
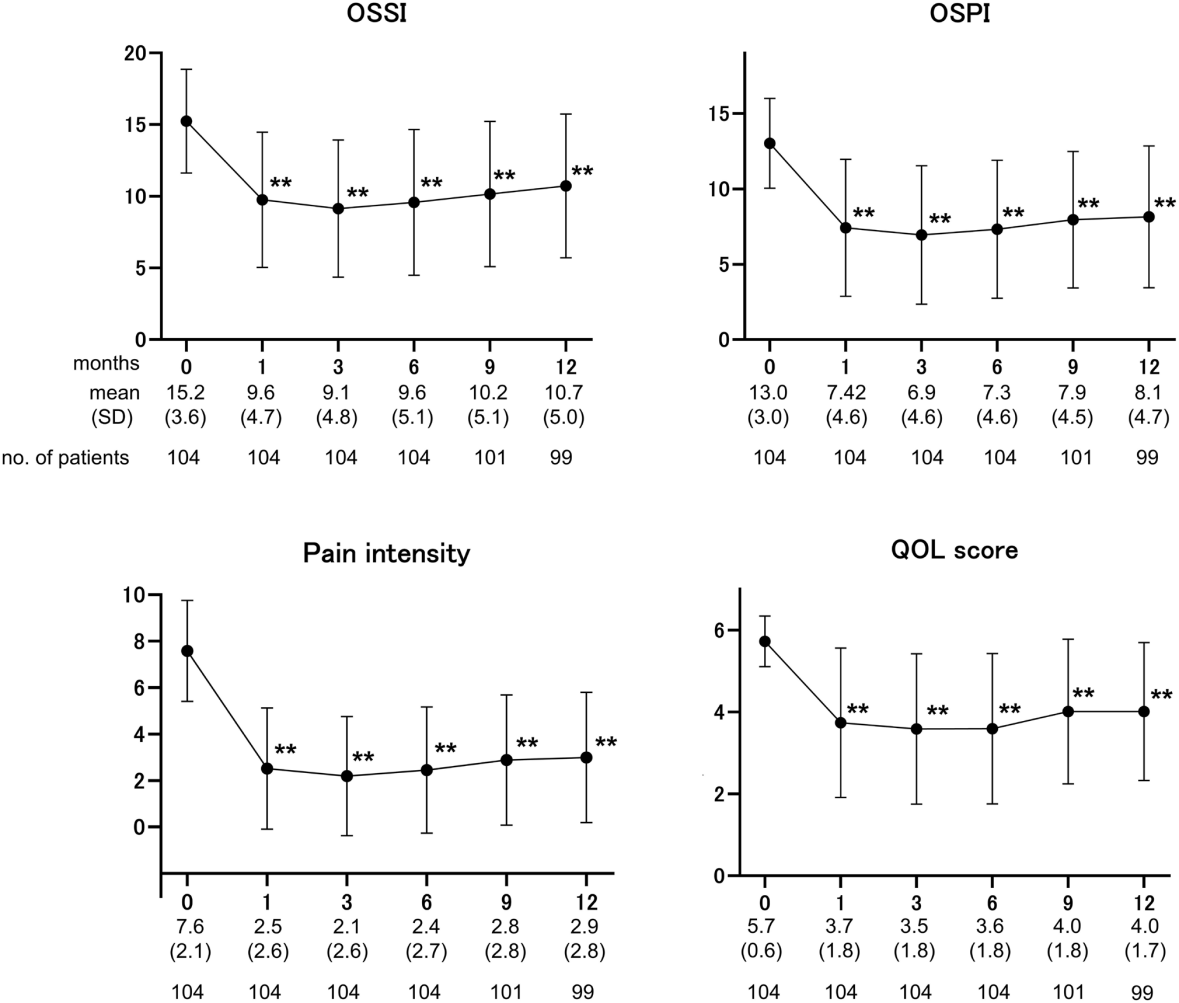
Symptom and QOL parameters during the 1‐year follow‐up. The OSSI/OSPI, QOL scores, and pain intensity improved significantly 1 month after surgery, and efficacy was maintained during the follow‐up period. Values are expressed as the mean ± standard deviation (SD). **p < 0.0001, statistically significant difference between each visit and baseline (0 months); two‐tailed pairwise comparison conducted using the Wilcoxon signed‐rank test and post hoc Bonferroni correction. OSSI/OSPI, O'Leary and Sant symptom index/O'Leary and Sant Problem Index; QOL, quality of life.

**FIGURE 5:**
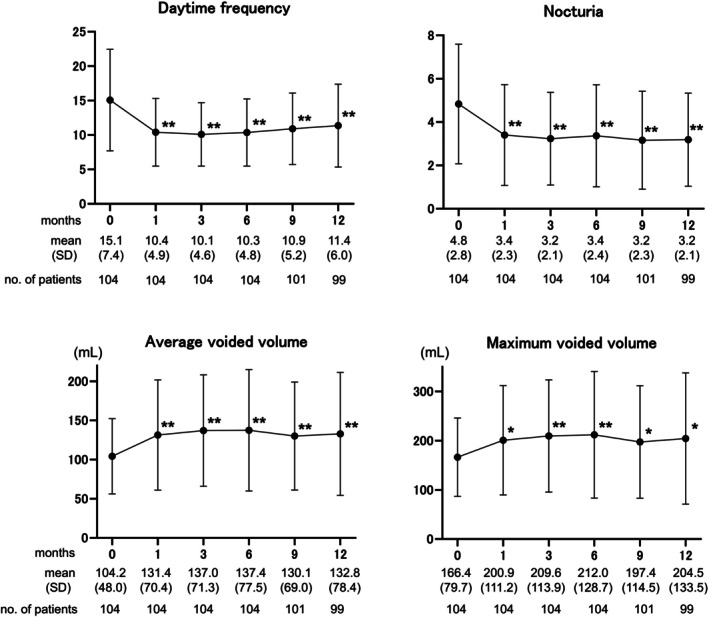
Urinary frequency and voided volume during the 1‐year follow‐up. Daytime frequency and nocturia were significantly reduced 1 month after surgery, in parallel with a significant increase in the average and maximum voided volume. *p < 0.001, **p < 0.0001; statistically significant difference between each visit and baseline (0 months); two‐tailed pairwise comparisons were conducted using the Wilcoxon signed‐rank test and post hoc Bonferroni correction. Values are expressed as the mean ± standard deviation (SD).

The overall response rates at 1, 3, 6, 9, and 12 months were 78.8%, 80.8%, 76.0%, 69.2%, and 57.7%, respectively (Figure 3). Compared to the baseline values, the OSSI/OSPI, QOL scores, and pain intensity decreased significantly at 1 month, and the efficacy was maintained over the course of a year (Figure 4). Likewise, daytime frequency and nocturia decreased significantly 1 month after surgery, along with a significant increase in AVV and MVV (Figure 5). No pretreatment parameters were predictive of the treatment response at 12 months (data not shown).

Of all patients, 42 remained symptom‐free at the end of the study, while the remaining 62 experienced relapse at the mean of 20.0 ± 12.9 months (range, 3–61) post‐surgery. Among these, five patients relapsed within 12 months. Of those five, three received subsequent treatment by 9 months, and the remaining two received by 12 months. Relapsed patients received a second session of surgery (*N* = 18), intravesical DMSO treatment (*N* = 34), or oral prednisolone (*N* = 15), or were treated with oral analgesia, including opioids, acetaminophen, pregabalin, and nonsteroidal anti‐inflammatory drugs (*N* = 37), as subsequent treatments.

In relapsed patients, AVV and MVV significantly decreased at the end of the study from the baseline values, as AVV decreased from 107.5 ± 49.9 mL to 93.3 ± 54.3 mL (*p* < 0.01), and the MVV decreased from 173.2 ± 83.3 mL to 144.3 ± 85.6 mL (*p* < 0.01), respectively. Meanwhile, in patients who maintained symptom‐free during the study, bladder capacity increased significantly at the end of the study from the baseline values, as the AVV increased from 101.2 ± 44.8 mL to 138.9 ± 77.8 mL (*p* < 0.01) and MVV increased from 158.9 ± 73.3 mL to 218.1 ± 119.1 mL (*p* < 0.01).

### 3.3 Safety of Endoscopic Surgery

One patient required reoperation for hemostasis on the first postoperative day because of arterial bleeding at the site of fulgurated bladder mucosa. One male patient developed distal urethral stenosis 6 months postoperatively, requiring catheter dilations at three follow‐up visits. No other patients experienced any postoperative complications.

## 4 Discussion

In the present study, endoscopic surgery comprising transurethral resection of Hunner lesions and concomitant bladder hydrodistension was found to significantly improve bladder pain and lower urinary tract symptoms, as well as increase bladder capacity and improve QOL in treatment‐naïve patients with HIC without causing any serious adverse events. Our surgery provided significant immediate relief from pelvic/urethral pain and lower urinary tract symptoms 1 month after the procedure, with benefits lasting over 30 months. These results align with, or exceed those of previous reports [15, 18], indicating that first‐line endoscopic surgery is a promising gold‐standard treatment option for patients with HIC.

A Hunner lesion is a hallmark clinicopathological feature of HIC, histologically characterized by urothelial denudation and subepithelial chronic inflammatory changes [5, 20]. Disruption of the urothelial barrier at the lesion site allows urinary irritants to directly stimulate afferent neurons within the subepithelial layers, resulting in irritative symptoms of HIC [4, 21]. Therefore, precise identification and resection of Hunner lesions is important for achieving favorable treatment outcomes [21]. Meanwhile, excessive fulguration of the bladder mucosa may adversely affect bladder capacity and morphology. When hydrodistension is employed prior to electrocautery of Hunner lesions, recognition of Hunner lesions could be compromised due to mucosal bleeding after bladder distension, potentially resulting in insufficient or excessive treatment of the lesions. To mitigate this challenge, we marked Hunner lesions circumferentially prior to hydrodistension to accurately delineate their extent. This approach may support the long‐term therapeutic results of our surgery and help prevent excessive resection of the bladder mucosa.

Although this study found that the efficacy of the surgery lasted for more than 30 months, more than half of the patients eventually relapsed and required repeated procedures or further treatment. With respect to this, repeating surgeries may lead to a decrease in bladder capacity. We previously reported that bladder capacity decreased by approximately 50 mL per single procedure of this surgery, and this loss was offset by 10 mL per single year the repeating session was postponed [22]. Given this possible risk of affecting bladder capacity, endoscopic surgery for Hunner lesions should be performed as few times as possible. Nonetheless, the findings of the present study suggest that the first session of this surgery may not affect bladder capacity, as indicated by the increased functional bladder capacity in patients who remained symptom‐free during the study period. The observed decrease in bladder capacity in relapsed patients seemed to be attributable to painful urgency and the need to void. However, there was a possibility that limiting treatment to only the initially identified Hunner lesions might have led to inadequate management of other inflamed bladder regions that contributed to patients' symptoms. Further long‐term observations by disease risk stratification, including the intensity of histological inflammation, condition of comorbid systemic autoimmune diseases, and extent of Hunner lesions, are warranted on the relationship of the frequency and extent of bladder mucosal fulguration with changes in bladder capacity.

Evidence is increasingly highlighting the potential of immunomodulatory therapies and anti‐inflammatory drugs as alternative or adjunct treatment options to endoscopic surgery in patients with HIC. Cyclosporine A and tacrolimus have been used in the treatment of HIC, showing to be effective in improving pain and urinary symptoms [13, 14, 23, 24, 25]. In addition, corticosteroid has also been examined for patients with HIC [12, 26]. Intravesical injection of triamcinolone has been widely used as a minimally invasive endoscopic treatment and shown to be effective in patients with HIC [11, 27, 28]. Recently, we reported that low‐dose oral prednisolone (7.5 mg/body) mitigated the intractable pain and urinary tract symptoms in patients with HIC who relapsed after the endoscopic surgery, without any significant adverse events related to corticosteroid use [12]. The results also indicated that corticotherapy did not affect bladder capacity; rather, it increased its functional capacity. The efficacy and safety of intravesical treatment with DMSO in patients with HIC have also been shown in a multicenter, double‐blind, randomized clinical trial and subsequent real‐world data in Japan [10, 29]. Both studies demonstrated that intravesical injections of DMSO, administered every 2 weeks for a total of six times over 12 weeks, improve pain and lower urinary tract symptoms, as well as increase bladder capacity in patients with HIC. These results led to the official approval of intravesical DMSO for the treatment of HIC in Japan.

A histological assessment of the affected bladder is crucial for the proper diagnosis and treatment of HIC. Although the histological criteria for diagnosing HIC have not yet been established, certain histological features can aid in its diagnosis. These include epithelial denudation, dense inflammatory infiltrates predominantly composed of lymphoplasmacytic cells frequently accompanied by lymph follicles/aggregates in subepithelial layers, and stromal edema and fibrosis [3, 5, 20]. At our institution, histological evaluation of bladder biopsy specimens obtained from both Hunner lesions and background non‐lesional areas at the initial session of endoscopic surgery is routinely performed to determine whether bladder tissue exhibits histological features characteristic of HIC and is eligible for future immunomodulatory therapies.

Taken together, our current therapeutic approach for HIC seeks to balance satisfactory treatment efficacy with preserving bladder capacity. Early intervention with immunomodulatory and anti‐inflammatory therapies using DMSO and oral corticosteroids following initial endoscopic surgery is particularly emphasized in patients who meet the pathological criteria and exhibit markedly reduced bladder capacity and/or pronounced bladder inflammation.

This study has several limitations. First, its retrospective nature and the potential effect of being conducted by the same surgeon (YA) limit its methodological quality. The lack of a control group also limited the interpretation of the efficacy of this surgery. Further prospective randomized placebo‐controlled studies comparing endoscopic surgery with immunomodulatory therapies for HIC are warranted.

In conclusion, our study indicates that transurethral resection of Hunner lesions combined with bladder hydrodistension is a viable initial treatment option for surgically treatment‐naïve patients with HIC with undetermined bladder pathology.

## Author Contributions

**Yoshiyuki Akiyama:** conceptualization, formal analysis, writing – original draft, investigation, methodology. **Kenichi Hashimoto:** investigation. **Aya Niimi:** writing – review and editing. **Yukio Homma:** writing – review and editing. **Haruki Kume:** writing – review and editing.

## Ethics Statement

The study protocol, including the use of an opt‐out methodology to obtain informed consent, was approved by the institutional review board of the University of Tokyo (approval no. 3124) and conformed to the provisions of the Declaration of Helsinki.

## Consent

The participants were informed of the study using generally accessible contact information. Written informed consent was obtained from all patients who chose to participate in the study. All procedures followed the appropriate guidelines.

## Conflicts of Interest

Yoshiyuki Akiyama and Haruki Kume are the Editorial Board members of the International Journal of Urology and the coauthors of this article. To minimize bias, they were excluded from all editorial decision‐making related to the acceptance of this article for publication. Otherwise, the authors have no relevant financial interests to disclose regarding the materials discussed in the manuscript.

## References

[1] Y. Homma , Y. Akiyama , J. H. Kim , et al., “Definition Change and Update of Clinical Guidelines for Interstitial Cystitis and Bladder Pain Syndrome,” Lower Urinary Tract Symptoms 16, no. 5 (2024): e12532. 39267358 10.1111/luts.12532

[2] Y. Homma , Y. Akiyama , H. Tomoe , et al., “Clinical Guidelines for Interstitial Cystitis/Bladder Pain Syndrome,” International Journal of Urology 27, no. 7 (2020): 578–589. 32291805 10.1111/iju.14234

[3] S. L. Johansson and M. Fall , “Clinical Features and Spectrum of Light Microscopic Changes in Interstitial Cystitis,” Journal of Urology 143, no. 6 (1990): 1118–1124. 2342171 10.1016/s0022-5347(17)40201-1

[4] Y. Akiyama , Y. Luo , P. M. Hanno , D. Maeda , and Y. Homma , “Interstitial Cystitis/Bladder Pain Syndrome: The Evolving Landscape, Animal Models and Future Perspectives,” International Journal of Urology 27, no. 6 (2020): 491–503. 32246572 10.1111/iju.14229 PMC7768977

[5] D. Maeda , Y. Akiyama , T. Morikawa , et al., “Hunner‐Type (Classic) Interstitial Cystitis: A Distinct Inflammatory Disorder Characterized by Pancystitis, With Frequent Expansion of Clonal B‐Cells and Epithelial Denudation,” PLoS One 10, no. 11 (2015): e0143316. 26587589 10.1371/journal.pone.0143316 PMC4654580

[6] Y. Akiyama , D. Maeda , H. Katoh , et al., “Molecular Taxonomy of Interstitial Cystitis/Bladder Pain Syndrome Based on Whole Transcriptome Profiling by Next‐Generation RNA Sequencing of Bladder Mucosal Biopsies,” Journal of Urology 202, no. 2 (2019): 290–300. 30865573 10.1097/JU.0000000000000234

[7] J. P. van de Merwe , “Interstitial Cystitis and Systemic Autoimmune Diseases,” Nature Clinical Practice. Urology 4, no. 9 (2007): 484–491. 10.1038/ncpuro0874 17823601

[8] Y. Akiyama , K. Sonehara , D. Maeda , et al., “Genome‐Wide Association Study Identifies Risk Loci Within the Major Histocompatibility Complex Region for Hunner‐Type Interstitial Cystitis,” Cell Reports Medicine 4, no. 7 (2023): 101114. 37467720 10.1016/j.xcrm.2023.101114 PMC10394254

[9] K. J. Ko , W. J. Cho , Y. S. Lee , J. Choi , H. J. Byun , and K. S. Lee , “Comparison of the Efficacy Between Transurethral Coagulation and Transurethral Resection of Hunner Lesion in Interstitial Cystitis/Bladder Pain Syndrome Patients: A Prospective Randomized Controlled Trial,” European Urology 77, no. 5 (2020): 644–651. 31959549 10.1016/j.eururo.2020.01.002

[10] N. Yoshimura , Y. Homma , H. Tomoe , et al., “Efficacy and Safety of Intravesical Instillation of KRP‐116D (50% Dimethyl Sulfoxide Solution) for Interstitial Cystitis/Bladder Pain Syndrome in Japanese Patients: A Multicenter, Randomized, Double‐Blind, Placebo‐Controlled, Clinical Study,” International Journal of Urology 28, no. 5 (2021): 545–553. 33580603 10.1111/iju.14505 PMC8247858

[11] M. G. Funaro , A. N. King , J. N. H. Stern , R. M. Moldwin , and S. Bahlani , “Endoscopic Injection of Low Dose Triamcinolone: A Simple, Minimally Invasive, and Effective Therapy for Interstitial Cystitis With Hunner Lesions,” Urology 118 (2018): 25–29. 29782887 10.1016/j.urology.2018.03.037

[12] Y. Akiyama , A. Niimi , A. Nomiya , et al., “Efficacy and Safety of Low‐Dose Oral Prednisolone for Patients With Refractory Hunner‐Type Interstitial Cystitis,” European Urology Open Science 56 (2023): 1–8. 37822513 10.1016/j.euros.2023.07.006 PMC10562155

[13] J. B. Forrest , C. K. Payne , and D. R. Erickson , “Cyclosporine A for Refractory Interstitial Cystitis/Bladder Pain Syndrome: Experience of 3 Tertiary Centers,” Journal of Urology 188, no. 4 (2012): 1186–1191. 22901569 10.1016/j.juro.2012.06.023

[14] P. C. Bosch , “A Randomized, Double‐Blind, Placebo‐Controlled Trial of Certolizumab Pegol in Women With Refractory Interstitial Cystitis/Bladder Pain Syndrome,” European Urology 74, no. 5 (2018): 623–630. 30072210 10.1016/j.eururo.2018.07.026

[15] S. W. Lee , W. B. Kim , K. W. Lee , et al., “Transurethral Resection Alone vs Resection Combined With Therapeutic Hydrodistention as Treatment for Ulcerative Interstitial Cystitis: Initial Experience With Propensity Score Matching Studies,” Urology 99 (2017): 62–68. 27720770 10.1016/j.urology.2016.09.038

[16] A. Niimi , A. Nomiya , Y. Yamada , et al., “Hydrodistension With or Without Fulguration of Hunner Lesions for Interstitial Cystitis: Long‐Term Outcomes and Prognostic Predictors,” Neurourology and Urodynamics 35, no. 8 (2016): 965–969. 26208131 10.1002/nau.22837

[17] A. Chennamsetty , I. Khourdaji , J. Goike , K. A. Killinger , B. Girdler , and K. M. Peters , “Electrosurgical Management of Hunner Ulcers in a Referral Center's Interstitial Cystitis Population,” Urology 85, no. 1 (2015): 74–78. 25440759 10.1016/j.urology.2014.09.012

[18] E. S. Lee , S. W. Lee , K. W. Lee , J. M. Kim , Y. H. Kim , and M. E. Kim , “Effect of Transurethral Resection With Hydrodistention for the Treatment of Ulcerative Interstitial Cystitis,” Korean Journal of Urology 54, no. 10 (2013): 682–688. 24175042 10.4111/kju.2013.54.10.682 PMC3806992

[19] J. H. Hillelsohn , S. Rais‐Bahrami , J. I. Friedlander , et al., “Fulguration for Hunner Ulcers: Long‐Term Clinical Outcomes,” Journal of Urology 188, no. 6 (2012): 2238–2241. 23083651 10.1016/j.juro.2012.08.013

[20] Y. Akiyama , Y. Homma , and D. Maeda , “Pathology and Terminology of Interstitial Cystitis/Bladder Pain Syndrome: A Review,” Histology and Histopathology 34, no. 1 (2019): 25–32. 30015351 10.14670/HH-18-028

[21] Y. Akiyama , A. Niimi , A. Nomiya , et al., “Extent of Hunner Lesions: The Relationships With Symptom Severity and Clinical Parameters in Hunner Type Interstitial Cystitis Patients,” Neurourology and Urodynamics 37, no. 4 (2018): 1441–1447. 29315774 10.1002/nau.23467

[22] Y. Akiyama , M. Zaitsu , D. Watanabe , et al., “Relationship Between the Frequency of Electrocautery of Hunner Lesions and Changes in Bladder Capacity in Patients With Hunner Type Interstitial Cystitis,” Scientific Reports 11, no. 1 (2021): 105. 33420263 10.1038/s41598-020-80589-3 PMC7794499

[23] J. Sairanen , T. Forsell , and M. Ruutu , “Long‐Term Outcome of Patients With Interstitial Cystitis Treated With Low Dose Cyclosporine A,” Journal of Urology 171, no. 6 Pt 1 (2004): 2138–2141. 15126772 10.1097/01.ju.0000125139.91203.7a

[24] I. M. Crescenze , B. Tucky , J. Li , C. Moore , and D. A. Shoskes , “Efficacy, Side Effects, and Monitoring of Oral Cyclosporine in Interstitial Cystitis‐Bladder Pain Syndrome,” Urology 107 (2017): 49–54. 28528859 10.1016/j.urology.2017.05.016 PMC5595648

[25] A. Vollstedt , L. Tennyson , K. Turner , et al., “Evidence for Early Cyclosporine Treatment for Hunner Lesion Interstitial Cystitis,” Female Pelvic Medicine & Reconstructive Surgery 28, no. 1 (2022): e1–e5. 34608034 10.1097/SPV.0000000000001108

[26] F. Soucy and M. Grégoire , “Efficacy of Prednisone for Severe Refractory Ulcerative Interstitial Cystitis,” Journal of Urology 173, no. 3 (2005): 841–843. 15711286 10.1097/01.ju.0000153612.14639.19

[27] O. O. Cardenas‐Trowers , A. G. Abraham , T. K. Dotson , B. A. Houlette , J. T. Gaskins , and S. L. Francis , “Bladder Instillations With Triamcinolone Acetonide for Interstitial Cystitis‐Bladder Pain Syndrome: A Randomized Controlled Trial,” Obstetrics and Gynecology 137, no. 5 (2021): 810–819. 33831942 10.1097/AOG.0000000000004348

[28] T. Jiang , X. Zhou , Z. Chen , et al., “Clinical Efficacy of Submucosal Injection of Triamcinolone Acetonide in the Treatment of Type II/III Interstitial Cystitis/Bladder Pain Syndrome,” BMC Urology 20, no. 1 (2020): 36. 32228552 10.1186/s12894-020-00597-3 PMC7106786

[29] Y. Akiyama , A. Niimi , A. Nomiya , et al., “Efficacy and Safety of Intravesical Dimethyl Sulfoxide Treatment for Patients With Refractory Hunner‐Type Interstitial Cystitis: Real‐World Data Postofficial Approval in Japan,” International Journal of Urology 31, no. 2 (2024): 111–118. 37817647 10.1111/iju.15320 PMC11524091

